# Glycosphingolipids are essential entry factors for non-influenza orthomyxoviruses

**DOI:** 10.1099/jgv.0.002315

**Published:** 2026-08-13

**Authors:** Lorena Lozano-Montalbá, Zoé Denis, Valérie Courgnaud, Jorge Moreno-García, Karim Majzoub, Rafael Sanjuán, Jérémy Dufloo

**Affiliations:** 1 Institute for Integrative Systems Biology, CSIC-Universitat de València, Paterna, València, Spain; 2 Institut de Génétique Moléculaire de Montpellier (IGMM), Univ Montpellier, CNRS, Montpellier, France

**Keywords:** glycosphingolipids, orthomyxoviruses, quaranjavirus, thogotovirus, UGCG, viral entry

## Abstract

Thogotoviruses and quaranjaviruses are arthropod-borne orthomyxoviruses that circulate widely in wildlife and domestic animals and include several zoonotic members. Despite their close phylogenetic relationship to influenza viruses, the mechanisms underlying their replication remain poorly understood, and the host factors mediating viral entry are unknown. Here, we performed genome-wide loss-of-function CRISPR-Cas9 screens using replication-competent recombinant vesicular stomatitis viruses expressing thogoto- and quaranjavirus glycoproteins to identify cellular determinants of viral entry. These screens identified the glycosphingolipid (GSL) biosynthesis pathway as a key regulator of viral entry, with the upstream enzyme uridine diphosphate (UDP)-glucose ceramide glucosyltransferase (UGCG) emerging as a central host entry factor. Pharmacological inhibition of UGCG impaired thogoto- and quaranjavirus entry. The importance of GSL biosynthesis for thogotovirus replication was further validated using multiple thogotovirus isolates. Together, our findings establish GSLs as critical host entry factors for non-influenza orthomyxoviruses and identify UGCG as a potential target for antiviral intervention.

## Introduction

The *Thogotovirus* and *Quaranjavirus* genera of the *Orthomyxoviridae* family comprise tick-borne viruses that have drawn increasing attention because several members can occasionally infect humans. Reported zoonotic infections include Quaranfil virus, identified in two children with febrile illness [1]; Bourbon virus, associated with febrile illness in the USA including one fatal case [2]; Oz virus (OZV), which caused a fatal infection in Japan [3, 4]; Thogoto virus (THOV), reported in several patients in Nigeria [5]; and Dhori virus (DHOV), detected following an accidental laboratory infection [6].

Despite these human cases, thogotoviruses are primarily maintained in wild mammals, including rodents, dromedaries and banded mongooses, and can also infect domestic species such as cattle and other livestock, whereas quaranjaviruses mainly circulate in birds [7–9]. Serological surveys have detected antibodies against THOV and DHOV in livestock and humans in Spain and Portugal [8, 10], suggesting active circulation in Europe. In addition, we and others have shown that thogoto- and quaranjavirus glycoproteins (GPs) can mediate entry into a broad range of mammalian cells, including human cells, underscoring their zoonotic potential [11, 12]. Defining the host determinants that support infection by these non-influenza orthomyxoviruses is, therefore, important for assessing spillover risk and guiding preparedness efforts.

Thogoto- and quaranjaviruses encode a single GP that mediates attachment, entry and membrane fusion [13]. GP-driven membrane fusion is pH-dependent [14], and entry is thought to occur via endocytosis [14, 15]. However, the mammalian entry factors exploited by these viruses remain unknown. Their broad cell tropism suggests reliance on a widely expressed host receptor and/or the capacity to engage alternative receptors. Notably, in contrast to influenza viruses, we have shown that sialic acids inhibit thogoto- and quaranjavirus entry, further supporting the idea that these viruses use as-yet-unidentified receptors [16].

Here, we sought to improve our understanding of how non-influenza orthomyxoviruses enter host cells. Using genome-wide clustered regularly interspaced short palindromic repeats (CRISPR) screens, followed by validation assays with viral pseudotypes and authentic viral isolates, we identified glycosphingolipid (GSL) biosynthesis as a key determinant of thogoto- and quaranjavirus entry. Our results, thereby, establish GSLs as critical host factors for non-influenza orthomyxoviruses.

## Results

### Genome-wide CRISPR forward genetic screens identify GSLs as candidate thogoto- and quaranjavirus entry factors

**Fig. 1.**
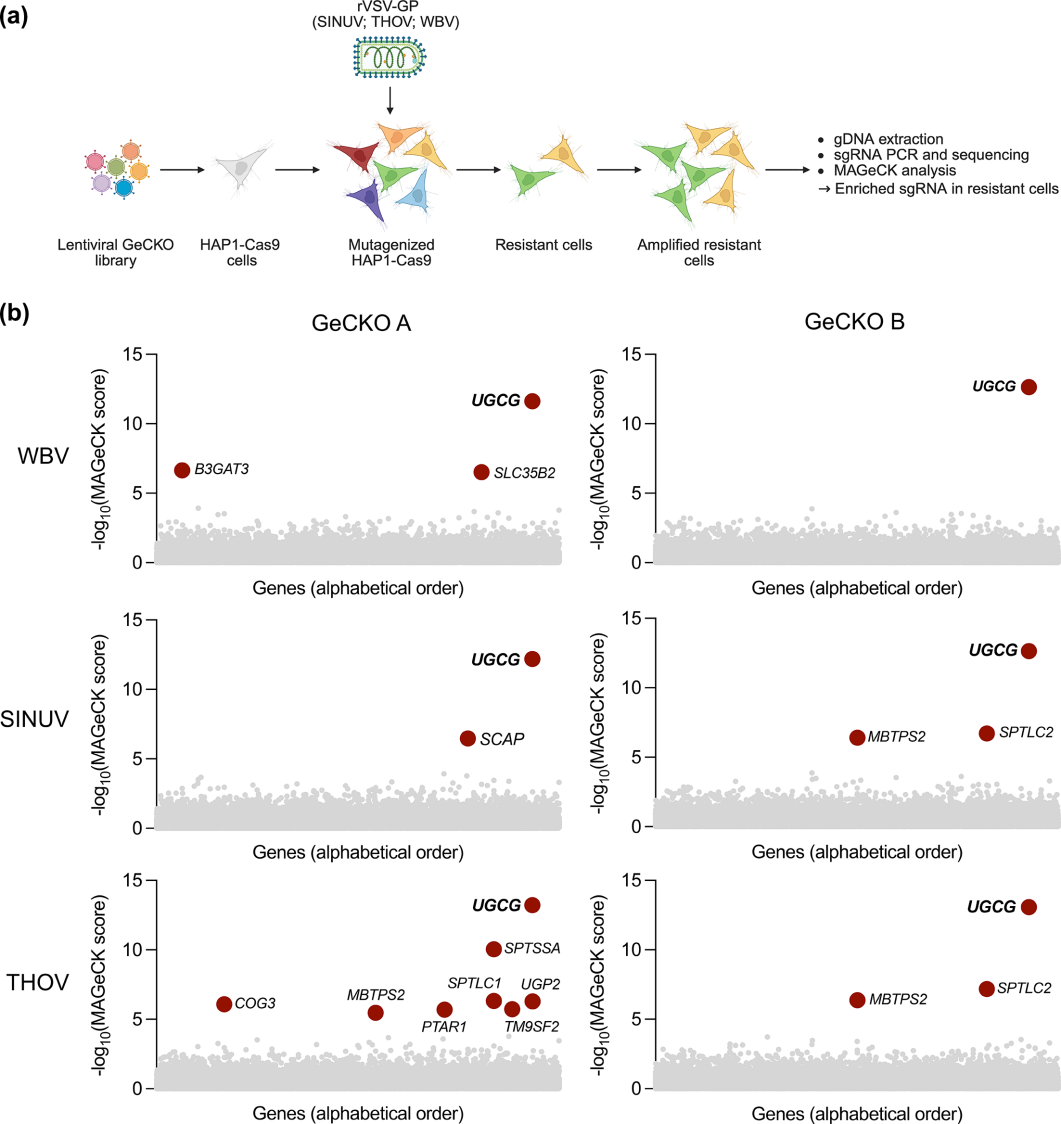
Genome-wide CRISPR screens identify GSLs as candidate entry factors for thogoto- and quaranjaviruses. (a) Schematic of the CRISPR screening workflow. HAP1-Cas9 cells were transduced with pooled lentiviral GeCKOv2 sub-libraries A and B and infected with recombinant vesicular stomatitis viruses (rVSV) expressing the GPs of WBV, SINUV or THOV. Cells resistant to virus-induced cytopathic effects were expanded, genomic DNA was isolated and enriched sgRNAs were identified by PCR amplification, NGS and computational analysis using the MAGeCK pipeline. (**b)** sgRNA enrichment in cells mutagenized with both GeCKOv2 sub-libraries (GeCKO **A and GeCKO **B) following infection with rVSV-WBV, rVSV-SINUV or rVSV-THOV. Data show log-transformed MAGeCK scores for all genes, displayed in alphabetical order. Significantly enriched genes are shown in red, with the top hit, UGCG, highlighted in bold. UGCG, UDP-glucose ceramide glucosyltransferase.

To identify host factors involved in thogoto- and quaranjavirus entry, we performed three genome-wide CRISPR-Cas9 loss-of-function forward genetic screens in haploid HAP1 cells [17]. We used replication-competent recombinant vesicular stomatitis viruses (rVSV) expressing GPs of three representative non-influenza orthomyxoviruses: THOV, the prototypical thogotovirus; Sinu virus (SINUV), a divergent thogotovirus found in mosquitoes [18]; and Wellfleet Bay virus (WBV), a quaranjavirus representative [19]. We used the GeCKOv2 library [20], which consists of two sub-libraries (A and B) comprising 123,411 single-guide RNAs (sgRNAs) targeting 19,050 genes. Cells mutagenized with both sub-libraries were infected with rVSV-WBV, rVSV-SINUV or rVSV-THOV. Cells resistant to virus-induced cytopathic effects were expanded, and sgRNAs from non-selected and resistant populations were amplified from genomic DNA by PCR. The PCR products were subjected to next-generation sequencing (NGS), and sgRNA enrichment in resistant cell populations was analysed using the MAGeCK pipeline [21] (Fig. 1a).

Strikingly, sgRNAs targeting the *UGCG* gene were consistently and highly enriched in cells resistant to rVSV bearing GPs of all three tested viruses, in both GeCKOv2 sub-libraries (−log_10_ MAGeCK scores>11 in all cases; Fig. 1b). Uridine diphosphate (UDP)-glucose ceramide glucosyltransferase [UGCG, also known as glucosylceramide (GlcCer) synthase] catalyses the first glycosylation step in GSL biosynthesis [22]. GSLs are a sub-class of glycolipids that are integral components of the plasma membrane and are involved in various processes such as cell adhesion and cell–cell interactions [23]. Notably, sgRNAs targeting additional genes directly or indirectly involved in GSL biosynthesis were also enriched, albeit to a lesser extent and not in all conditions. sgRNAs targeting *SPTLC1*, *SPTSSA*, *TM9SF2* and UDP-glucose pyrophosphorylase 2 (*UGP2*) were enriched in rVSV-THOV-resistant cells (sub-library GeCKO A), whereas sgRNAs targeting *SPTLC2* were enriched in rVSV-SINUV- and rVSV-THOV-resistant cells (sub-library GeCKO B). SPTSSA, SPTLC1 and SPTLC2 are subunits of serine palmitoyltransferase, which catalyses the first step of sphingolipid biosynthesis [24]. TM9SF2 has recently been shown to contribute to GSL biosynthesis by regulating the activity of Gb3 synthase, another enzyme in the pathway [25]. Finally, UGP2 produces UDP-glucose, a sugar donor required for multiple glycosylation reactions, including glycolipid synthesis [26]. Altogether, these results suggest that GSLs are essential entry factors for thogoto- and quaranjaviruses. Because UGCG emerged as a recurrent high-scoring hit across all screens and is a key upstream enzyme in the GSL biosynthesis pathway, we focused on UGCG to further investigate the role of GSLs in thogoto- and quaranjavirus entry and replication.

### UGCG is required for thogoto- and quaranjavirus entry

**Fig. 2.**
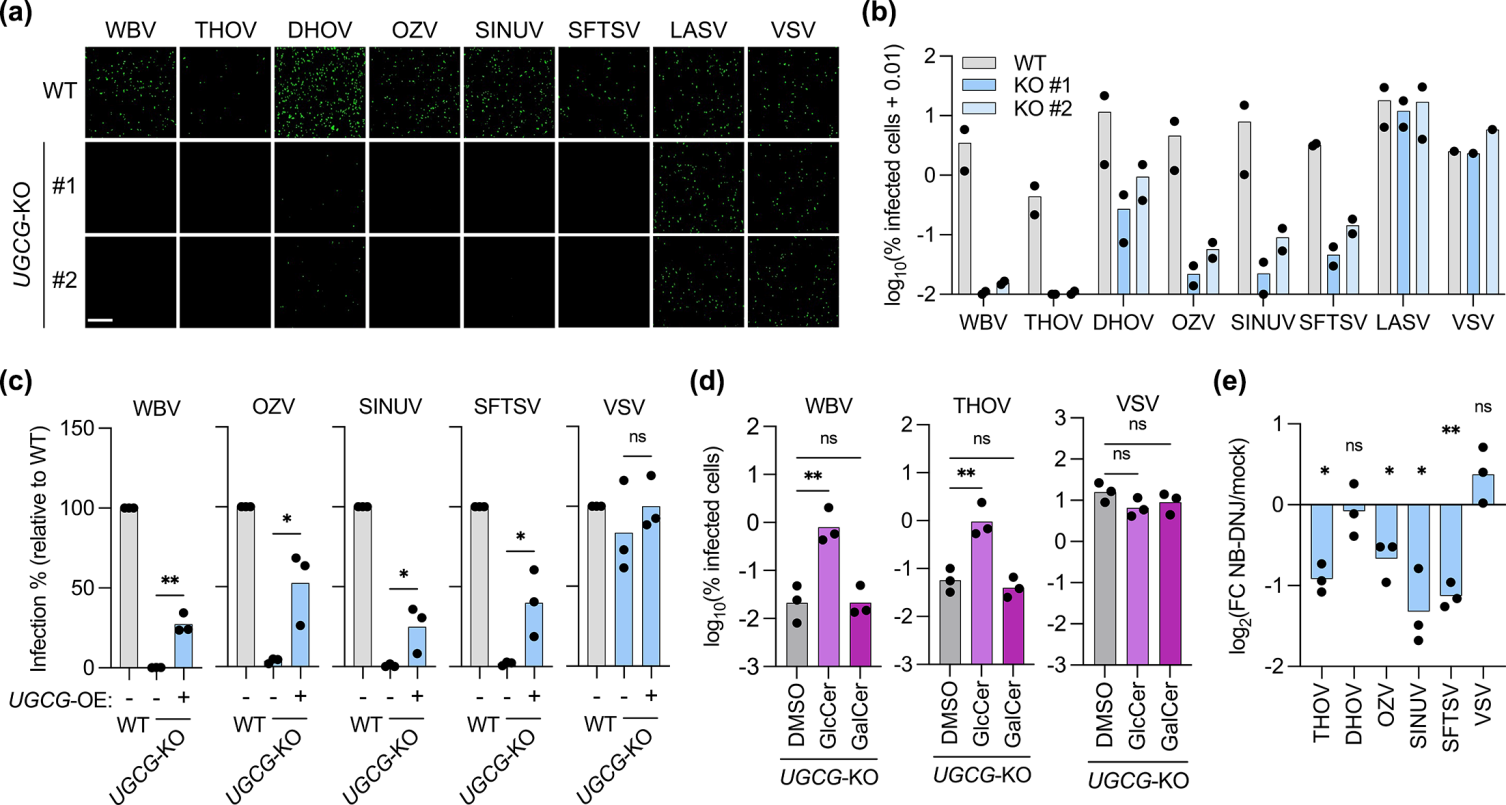
UGCG-mediated GSL synthesis is necessary for thogotovirus and quaranjavirus entry. (a) Representative fluorescence microscopy images of WBV, THOV, DHOV, OZV, SINUV, SFTSV, LASV and VSV pseudotype infection in WT HEK293T cells (top row) and in two independent *UGCG*-KO clones (clones #1 and #2; middle and bottom rows). Scale bar, 400 µm.** (b)** Percentage of infection by the indicated pseudotypes in WT HEK293T cells and in the two *UGCG*-KO clones. (**c)** *UGCG*-KO HEK293T cells were transfected with either an empty vector or a *UGCG* expression construct (*UGCG*-OE) prior to infection with the indicated viral pseudotype. Infection percentages were normalized to infection levels in WT cells. Statistical significance was assessed using an unpaired t-test. (**d)** *UGCG*-KO HEK293T cells were treated with 50 µM glucosylceramide (GlcCer), galactosylceramide (GalCer) or DMSO vehicle control prior to infection with the indicated viral pseudotype. Log-transformed infection percentages are shown. Statistical significance was assessed using one-way ANOVA followed by Dunnett’s multiple-comparisons test. (**e)** HEK293T cells were treated with the UGCG inhibitor NB-DNJ prior to infection with the indicated viral pseudotype. Log-transformed fold changes in infection percentages relative to mock-treated cells are shown. Statistical significance was determined using a one-sample t-test, with values compared to zero. In all panels, each dot represents an independent experiment (*n*=2–3), bars indicate the mean and statistical significance was defined as follows: *P<0.05; **P<0.01; ns, not significant.

To confirm the importance of UGCG in thogoto- and quaranjavirus entry, we used a CRISPR-Cas9-mediated knockout (KO) approach to generate two independent *UGCG*-KO single-cell clones in HEK293T cells. *UGCG*-KO cells were then infected with single-cycle VSV pseudotypes bearing the GPs from a broader panel of non-influenza orthomyxoviruses, including WBV, THOV, SINUV, DHOV and OZV. VSV pseudotyped with GPs from severe fever with thrombocytopenia syndrome virus (SFTSV), Lassa virus (LASV) or VSV-G GP were also included to confirm specificity. SFTSV (family *Phenuiviridae*) served as a positive control, as previous studies have demonstrated a role for UGCG in SFTSV entry [27, 28]. LASV (family *Arenaviridae*) and VSV (family *Rhabdoviridae*) GPs served as negative controls, as their entry is not expected to depend on GSLs. In both *UGCG*-KO HEK293T clones, UGCG deficiency almost completely inhibited entry mediated by all tested thogoto- and quaranjavirus GPs, as well as by the SFTSV GP (Fig. 2a, b). In contrast, entry mediated by VSV-G or LASV-GP was unaffected in *UGCG*-KO cells, indicating that GSL dependence is specific to thogoto- and quaranjavirus GP-mediated entry. Rescue of UGCG expression by transient overexpression in *UGCG*-KO cells restored the infectivity of WBV, OZV and SINUV pseudotypes without affecting VSV-G-mediated entry (Fig. 2c). Together, these results confirm that UGCG is critical for the entry of non-influenza orthomyxoviruses.

### UGCG-mediated GSL synthesis is critical for the entry of non-influenza orthomyxoviruses

We next sought to determine whether the effect of UGCG on thogoto- and quaranjavirus entry was indeed linked to its known role in GSL biosynthesis. UGCG catalyses the formation of GlcCer, the precursor of many GSL species. Supplementation of the cell culture medium with GlcCer rescued the infectivity of WBV and THOV pseudotypes in *UGCG*-KO cells (38- and 19-fold increases, respectively), without affecting VSV-G-mediated entry (Fig. 2d). In contrast, supplementation with galactosylceramide (GalCer), a GSL whose synthesis is independent of UGCG, had no effect on viral entry. Importantly, inhibition of UGCG-mediated GSL biosynthesis with *N*-butyldeoxynojirimycin (NB-DNJ, also known as miglustat), an FDA-approved drug used to treat Gaucher’s disease, resulted in a~2-fold reduction in the entry of THOV, OZV and SINUV pseudotypes (Fig. 2e). This reduction was comparable to that observed for SFTSV, for which a previous study reported a similar degree of entry inhibition by NB-DNJ [27]. In contrast, VSV-G-mediated entry was unaffected by NB-DNJ treatment, indicating that the requirement for UGCG-dependent GSL synthesis is specific to thogotovirus GP-mediated entry. DHOV GP-mediated entry was not decreased by NB-DNJ treatment, suggesting that this thogotovirus species is less dependent on GSLs for entry. Collectively, our findings reveal that UGCG-mediated GSL biosynthesis is required for the entry of non-influenza orthomyxoviruses.

### GSL synthesis is essential for infection by authentic thogotovirus isolates

**Fig. 3.**
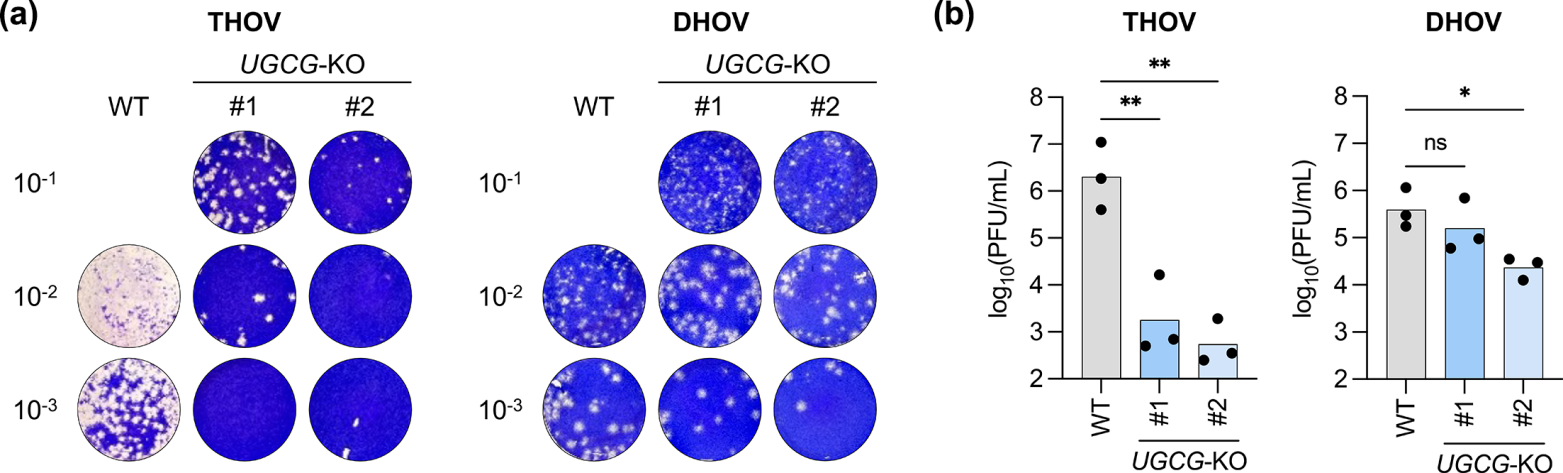
UGCG deficiency inhibits thogotovirus replication. (a) WT and two independent *UGCG*-KO HEK293T clones were infected with THOV or DHOV. Viral production in the supernatant was measured at 48 h post-infection by plaque assay on Vero cells. Representative images of the plaque assay are shown, with supernatant dilutions indicated on the left. (**b)** Log-transformed viral titres expressed as plaque-forming units per millilitre (PFU/mL). Each data point represents an independent experiment (*n*=3), and bars indicate the mean. Statistical significance was determined using an ordinary one-way ANOVA with Dunnett’s multiple-comparisons test. *P<0.05; **P<0.01; ns, not significant.

Finally, we sought to determine the role of GSLs in thogotovirus replication using authentic viral isolates. We used THOV (strain SiAr/126/72) and DHOV (strain India/1313/6) as representatives of the two principal phylogroups within the *Thogotovirus* genus [29]. WT and *UGCG*-KO HEK293T cells were infected with both viruses, and viral production at 48 h post-infection was quantified by plaque assay titration on Vero cells (Fig. 3a). *UGCG* KO strongly inhibited THOV infection in both *UGCG*-KO clones, with up to a 5,300-fold reduction in viral titres (Fig. 3b). In contrast, DHOV infection was significantly inhibited in only one of the two *UGCG*-KO clones, with a 21-fold reduction in viral titres. The lower susceptibility of DHOV infection to *UGCG* KO is consistent with the reduced sensitivity of DHOV entry to UGCG inhibition described above (Fig. 2e). Altogether, our results confirm the crucial role of GSL biosynthesis in thogotovirus infection, while revealing differences in susceptibility among individual viral species.

## Discussion

Although they belong to the same family as influenza viruses, thogoto- and quaranjaviruses have received far less attention, and the mechanisms underlying their replication remain poorly defined, despite their active circulation and zoonotic potential. This study aimed to identify host entry factors for non-influenza orthomyxoviruses. Using genome-wide loss-of-function CRISPR screens, we identified UGCG-dependent GSL biosynthesis as a key determinant of thogoto- and quaranjavirus entry. The importance of GSLs in non-influenza orthomyxovirus entry and replication was confirmed using different pseudotyped viruses and authentic viral isolates. An important question arising from our findings is whether thogoto- and quaranjaviruses similarly exploit GSLs in their natural tick hosts. Although glycobiology in tick–virus interactions remains understudied, ticks encode a *UGCG* homologue and produce GSLs [30], raising the possibility that, as in mammalian cells, UGCG-dependent GSL synthesis might also contribute to viral infection and transmission in the arthropod vector.

UGCG is an intracellular glycosyltransferase predominantly localized to the early secretory pathway (endoplasmic reticulum/Golgi) [31], whereas thogotovirus entry is believed to occur within acidified endosomes [14, 15]. Moreover, the UGCG product GlcCer is generally considered a relatively low-abundance, transient biosynthetic intermediate that is rapidly converted into more complex GSLs. Therefore, it is unlikely that UGCG or GlcCer themselves directly enable viral entry. Instead, GlcCer is further modified into a wide array of structurally and functionally distinct GSL species. A more likely hypothesis is that one or more of these downstream GSLs, rather than GlcCer itself, facilitate viral entry. However, the precise mechanism remains unknown. Possible hypotheses include that downstream GSL species present at the plasma membrane or within endosomal membranes act as attachment factors or co-receptors, promote the membrane environment required for receptor engagement or fusion, or regulate endocytic trafficking. Our experiments do not distinguish between these possibilities, and identifying both the specific GSL species involved and the precise step of viral entry they influence will require further investigation.

Although we observe a broad effect of *UGCG* KO on thogoto- and quaranjavirus entry, experiments with authentic viral isolates show that DHOV was much less affected by GSL biosynthesis disruption than THOV (Fig. 3). The underlying reasons for this difference would be worth investigating in future studies. Moreover, in this study, we did not determine whether authentic quaranjavirus isolates also require GSLs for replication. A better mechanistic understanding of how non-influenza orthomyxoviruses exploit GSLs for entry will help explain species- or strain-specific differences in GSL dependence.

GSLs have been implicated in the entry of numerous viruses. Several non-enveloped viruses co-opt GSLs as attachment factors or receptors, including SV40 (GM1a), rotavirus (HBGA), BK virus (GD1b and GT1b) and norovirus (HBGA) [32–36]. Similarly, several enveloped viruses depend on GSLs for entry, including influenza virus, SARS-CoV-2, Sindbis virus and several members of the *Phenuiviridae* family, such as SFTSV, Heartland virus and Uukuniemi virus [27, 28, 37–41]. Given this broad involvement of GSLs in viral entry, inhibition of GSL biosynthesis via UGCG represents a promising broad-spectrum antiviral strategy. Notably, some UGCG inhibitors are already FDA-approved for other applications (e.g. NB-DNJ for treatment of Gaucher disease) and have demonstrated antiviral activity *in vitro* against several viruses, including Sindbis virus [41], SARS-CoV-2 and influenza viruses [39]. Consistent with these observations, our data show that NB-DNJ also impairs thogotovirus entry, suggesting that such approved UGCG inhibitors could therefore be readily repurposed as antiviral compounds, particularly during a thogoto- or quaranjavirus emergence event in humans.

## Methods

### Cells and viruses

HEK293T, BHK-G43 [42] (a kind gift of Dr. Gert Zimmer), BHK-21 and Vero cells were grown in Dulbecco's modified Eagle medium (DMEM; Gibco) supplemented with 10% fetal bovine serum (FBS; Gibco), 250 ng ml^−1^ amphotericin B (Gibco), 10 units/ml penicillin and 10 µg ml^−1^ streptomycin (Gibco) at 37 °C and 5% CO_2_. HEK293T-Cas9 cells were selected with blasticidin (10 µg ml^−1^, Gibco). HEK293T-Cas9-UGCG-KO cells were selected with blasticidin (10 µg ml^−1^; Fisher) and puromycin (2 µg ml^−1^; Fisher). HAP1 cells were grown in Iscove’s Modified Dulbecco’s Medium (Gibco) supplemented with 10% FBS, 10 units/ml penicillin and 10 µg ml^−1^ streptomycin at 37 °C and 5% CO_2_. Mycoplasma contamination was regularly discarded by PCR. THOV/SiAr/126/72 and DHOV/India/1313/6 isolates [29] are kind gifts of Pr. Georg Kochs (Universitätsklinikum Freiburg, Germany) and were propagated in BHK-21 cells.

### Generation of GP-expressing recombinant VSV

The WT WBV, SINUV and THOV GP sequences were cloned using HiFi assembly (New England Biolabs) into a VSV cDNA infectious clone lacking the VSV-G gene. Recovery of GP-expressing recombinant VSV (rVSV-GP) from the cDNA clone was performed as previously described [43]. Briefly, BHK-G43 cells (BHK-21 cells engineered to express the VSV GP upon mifepristone induction) were co-transfected with pVSVeGFP-ΔG-GP (a plasmid encoding the viral genome, an eGFP fluorescent reporter and the desired viral GP), helper plasmids encoding VSV P, N and L and the T7 RNA polymerase using lipofectamine 3000 (Invitrogen™). Medium was then replaced with complete DMEM with 10% FBS and 10 nM mifepristone to induce VSV-G expression, and cells were placed at 37 °C for 2–4 days. Supernatants from GFP-positive cells were harvested, clarified by centrifugation at 2,000 *g* for 10 min and used to infect normal, non-VSV-G expressing BHK-21 cells to remove residual VSV-G. After 1 h of infection at 37 °C, inoculum was removed, cells were washed with pre-warmed PBS five times and incubated in DMEM containing 2% FBS and 25% anti-VSV-G monoclonal antibody. Supernatants containing recombinant VSV encoding the glycoproteins of interest and cleaned from VSV-G were harvested 24–48 hpi, cleared by centrifugation as before and stored at −80 °C.

### Genome-wide haploid CRISPR-Cas9 KO screen

HAP1 cells stably expressing Cas9 were generated by lentiviral transduction (Addgene #52962 lentiCas9-Blast plasmid) and selected with 10 µg ml^−1^ blasticidin. HAP1-Cas9 cells were then transduced with lentiviral vectors encoding the GeCKOv2 sub-libraries A and B (Addgene # 1000000049) independently at a multiplicity of infection (MOI) of ~0.3, expanded and selected with 500 ng ml^−1^ puromycin (InvivoGen). A total of 60×10^6^ HAP1-Cas9 cells from each sub-library were subsequently infected with rVSV-GP. Cells resistant to virus-induced cytopathic effects were expanded for at least 1 week, after which resistant cells were pelleted and genomic DNA was isolated using the QIAamp DNA Blood Maxi Kit (Qiagen). Genomic DNA from unselected control cells was extracted in parallel. gRNA-containing fragments were amplified by PCR and sequenced on an Illumina NovaSeq X Series platform (Illumina). gRNAs enriched in selected cells were identified using the MAGeCK pipeline [21]. No new sequence data were generated as part of this work.

### Generation of CRISPR-Cas9 single-KO cell lines

A single guide RNA (sgRNA) targeting UGCG (5′-GCCTTACGTAGCAGACAGAC-3′) was cloned into the pRDA_118 vector (Addgene #133459) using Esp3I restriction. sgRNA-expressing lentiviruses were produced by transfecting HEK293T cells with a 1 : 1 : 1 ratio of the pRDA_118 sgRNA vector, the psPAX2 lentiviral packaging plasmid (Addgene #12260) and the pVSV-G GP plasmid. Cas9-expressing HEK293T cells, generated by lentiviral transduction with the pLX_311-Cas9 vector (Addgene #96924), were subsequently transduced with the sgRNA-expressing lentiviral vector, selected with puromycin and single-cell cloned by limiting dilution. KO efficiency was confirmed in two independent clones using Tracking of Indels by DEcomposition (TIDE) analysis [44]. One clone contained -2,–1 and +1 bp indels, whereas the second clone harboured −4 and +1 bp indels.

### VSV pseudotype production and infection

VSV pseudotypes bearing the THOV, DHOV, OZV, SINUV, WBV, LASV and SFTSV GPs were generated as previously described [12]. Briefly, HEK293T cells were transfected with plasmids encoding the respective GPs and, the following day, infected with VSVΔG+G, a virus lacking its native G GP gene and complemented in trans with VSV G. Cells were thoroughly washed with PBS, and pseudotyped viruses were harvested 24 h post-infection. VSV-G pseudotypes bearing the VSV G GP were produced as controls. Pseudotypes were mixed at a 1 : 1 ratio with an anti-VSV-G monoclonal antibody and incubated for 20 min at 37 °C to neutralize residual VSV-G before infection of WT and *UGCG*-KO HEK293T cells. After 18–24 h, plates were imaged using the Incucyte SX5 Live-Cell Analysis System (Sartorius). The percentage of infected cells was determined by calculating the ratio of GFP-positive area to total cell confluence, both quantified automatically using Incucyte Analysis software (v2022B Rev2).

### UGCG rescue

WT and *UGCG*-KO HEK293T cells were transfected in suspension with the codon-optimized UGCG coding sequence (RefSeq NM_003358.3) cloned into the pcDNA3.1 vector (Genscript) or with an empty vector control using Lipofectamine 3000 (Invitrogen), according to the manufacturer’s instructions. Forty-eight hours post-transfection, cells were infected with the viral pseudotypes, as described above.

### Sphingolipid treatment and pseudotype infection

*UGCG*-KO HEK293T cells were seeded in 96-well plates to achieve 90% confluence on the day of infection. The following day, the culture medium was replaced with DMEM supplemented with 10% FBS and antibiotics containing either 50 µM GlcCer [C16 Glucosyl(β) Ceramide (d18 : 1/16 : 0); Avanti, 860539P], 50 µM GalCer [C16 Galactosyl(β) Ceramide (d18 : 1/16 : 0); Avanti, 860521P] or an equivalent volume of DMSO as vehicle control. After 24 h, treated cells were infected with the viral pseudotypes, as described above.

### UGCG inhibition and pseudotype infection

HEK293T cells were seeded in 96-well plates to achieve 90% confluence on the day of infection. The following day, the culture medium was replaced with DMEM supplemented with 10% FBS and antibiotics containing either 100 µM NB-DNJ (Sigma-aldrich, B8299) or an equivalent volume of sterile Milli-Q water as vehicle control. After 24 h, treated cells were infected with the viral pseudotypes, as described above.

### Authentic THOV and DHOV infections

WT and *UGCG*-KO HEK293T cells were infected with THOV or DHOV at an MOI of 0.001. After 1.5 h at 37 °C, the inoculum was removed, cells were washed three times with PBS and DMEM supplemented with 1% FBS and antibiotics was added. After 48 h at 37 °C, viral production in the supernatant was quantified by plaque assay on Vero cells. Briefly, Vero cells at ~90% confluence in 12-well plates were infected with serial dilutions of the virus for 1.5 h at 37 °C. The inoculum was then removed and replaced with fresh culture medium containing 0.6% agar. After 3 days, cells were fixed with 3.7% formaldehyde and stained with 0.1% crystal violet. Plaques were counted and viral titres were calculated and expressed as plaque-forming units per millilitre (p.f.u./ml).

### Statistics

Statistics were performed in GraphPad Prism v.10. All details about statistical tests can be found in the figure legends or in the main text.

## Abbreviations

CRISPR: Clustered Regularly Interspaced Short Palindromic Repeats
DHOV: Dhori virus
DMEM: Dulbecco's modified Eagle medium
DMSO: Dimethyl sulfoxide
FBS: fetal bovine serum
GalCer: galactosylceramide
GlcCer: glucosylceramide
GP: glycoprotein
GSL: glycosphingolipid
KO: knockout
LASV: Lassa virus
MOI: multiplicity of infection
NB-DNJ: *N*-butyldeoxynojirimycin
NGS: next-generation sequencing
OZV: Oz virus
PCR: polymerase chain reaction
rVSV: replication-competent recombinant vesicular stomatitis viruses
rVSV-GP: GP-expressing recombinant VSV
sgRNAs: single-guide RNAs
SINUV: Sinu virus
THOV: Thogoto virus
TIDE: Tracking of Indels by DEcomposition
UGCG: UDP-glucose ceramide glucosyltransferase
UGP2: UDP-glucose pyrophosphorylase 2
WBV: Wellfleet Bay virus
WT: wild type
